# Prediction of fall risk among community-dwelling older adults using a wearable system

**DOI:** 10.1038/s41598-021-00458-5

**Published:** 2021-10-25

**Authors:** Thurmon E. Lockhart, Rahul Soangra, Hyunsoo Yoon, Teresa Wu, Christopher W. Frames, Raven Weaver, Karen A. Roberto

**Affiliations:** 1 School of Biological and Health Systems Engineering, Arizona State University, Tempe, AZ 85281 USA; 2 Crean College of Health and Behavioral Sciences, Chapman University, Irvine, CA 92618 USA; 3 Fowler School of Engineering, Chapman University, Orange, CA 92866 USA; 4 Industrial Engineering, Yonsei University, Seoul, 03722 Korea; 5 School of Computing, Informatics, Decision Systems Engineering, Arizona State University, Tempe, AZ 85287 USA; 6 ASU-Mayo Center for Innovative Imaging, Arizona State University, Tempe, AZ 85287 USA; 7 Department of Human Development, College of Agricultural, Human, and Natural Resource Sciences, Washington State University, Pullman, WA 99164 USA; 8 Center for Gerontology, Virginia Polytechnic Institute and State University, Blacksburg, VA 24061 USA

**Keywords:** Health care, Geriatrics, Engineering, Biomedical engineering, Computational biology and bioinformatics, Classification and taxonomy

## Abstract

Falls are among the most common cause of decreased mobility and independence in older adults and rank as one of the most severe public health problems with frequent fatal consequences. In the present study, gait characteristics from 171 community-dwelling older adults were evaluated to determine their predictive ability for future falls using a wearable system. Participants wore a wearable sensor (inertial measurement unit, IMU) affixed to the sternum and performed a 10-m walking test. Measures of gait variability, complexity, and smoothness were extracted from each participant, and prospective fall incidence was evaluated over the following 6-months. Gait parameters were refined to better represent features for a random forest classifier for the fall-risk classification utilizing three experiments. The results show that the best-trained model for faller classification used both linear and nonlinear gait parameters and achieved an overall 81.6 ± 0.7% accuracy, 86.7 ± 0.5% sensitivity, 80.3 ± 0.2% specificity in the blind test. These findings augment the wearable sensor's potential as an ambulatory fall risk identification tool in community-dwelling settings. Furthermore, they highlight the importance of gait features that rely less on event detection methods, and more on time series analysis techniques. Fall prevention is a critical component in older individuals’ healthcare, and simple models based on gait-related tasks and a wearable IMU sensor can determine the risk of future falls.

## Introduction

Falls are one of the leading causes of injury and injury-related deaths among older adults^1^. Approximately 30% of adults over 65 years of age fall each year, in which almost 50% will likely fall more than once^2–4^. The consequence of falls are devastating, resulting in injuries^4^, reduced activity levels^5–7^, reduced quality of life^8,9^, increased fear of falling^5^, and ultimately, death ^5,10,11^. In 2014, 2.8 million nonfatal fall injuries were treated in emergency departments, and approximately 800,000 of these patients were subsequently hospitalized in the United States^1^. The direct-care costs of fall related-injuries and fatalities in 2015 was approximately $50.0 billion per year in the United States alone^69^. As such, predicting fall risk is imperative to provide an amenable intervention, however rates of falls resulting in injury have not been reported to have declined^12,13^.

Fall risk assessment is a powerful tool that aids in determining the risk of falls for a given individual in order to provide early diagnosis and treatment to reduce or prevent future falls. Traditionally, clinical fall risk assessments among older adults consist of questionnaires or functional assessments of posture and gait^14,15^; however, these methods are subjective and qualitative^14,16^. Whole-body motion analysis^17^, ground reaction forces^18^, and muscle activations^19^ have been utilized to develop objective and quantitative measures for fall risk assessment. However, these methods are not economically viable and take time to setup. This limits the applicability of these methods in obtaining fall risk characteristics. In that regard, wearable mobile sensors have several advantages over traditional laboratory-based systems for being portable and allowing for unobtrusive data gathering in non-laboratory environments. Wearable sensors, such as inertial measurement units (IMU), are able to monitor human movements as they respond to both frequency and intensity of movements, and measure both gravitational acceleration and acceleration due to body movement^20,21^. Additionally, a small wearable sensor can capture individuals’ activity during the daily living task (e.g., walking) performed in a real-world rather than simulated activities in laboratories.

Among community-dwelling older adults, falls are likely to occur during ambulation^22^. Previous studies have shown that trunk accelerations play a critical role in obtaining stable gait^23–26^ because trunk movement dynamics regulate gait-related oscillations in all three directions^25^. Some linear measures extracted from the trunk accelerometer (e.g., temporal parameters) have indicated that variability in the signal is associated with fall risk^27^. However, aging may induce subtle impairments in gait and without obviously detectable unsteadiness. Thus, nonlinear measures which are able to detect the hidden, subtle characteristics of aging in detrimental effects on locomotor control are also used (e.g., multiscale entropy, MSE and recurrence quantification analysis, RQA)^28–30^. Using the nonlinear methods shifts focus from the amount of variability in the signal to the structure and/or organization of variability. This fundamental difference may explain that nonlinear measures of kinematic trunk signals reveal subtle temporal properties of signals which are not detected in fallers through the traditional linear approach^31–33^. Furthermore, trunk movement during walking have been linked with age-related gait dysfunction^66,67^ and the risk of falls^68^ and, consequently, pathology in identifying fall risk in idiopathic fallers^34,35^.

Currently, the debate in fall literature is whether fall risk prediction is feasible such that new interventions could target modifiable risk factors^36^. In this study, we developed a random forest (RF) classification model using features extracted from linear and nonlinear gait parameters for fall risk prediction from a large set of a wearable (i.e., IMU) data during walking from community-dwelling older adults. This study focused on two main areas of work to develop tools of clinical value in fall prediction: (1) Developing a fall prediction model using RF framework using 127 participants’ walking data utilizing linear and nonlinear optimal gait features to obtain clinically relevant information using wearable sensor technology. (2) Testing the generalization and robustness of the predictive model on 44 community-dwelling older adults with six months follow up of their fall history. We hypothesized that both linear and nonlinear gait characteristics using only a few steps (i.e., 10-m walking condition) assessed by a wearable IMU sensor could aid in the prediction of fall risk among community-dwelling older adults.

## Results

In the present study, gait characteristics from 171 community-dwelling older adults were evaluated to determine their predictive ability for future falls. Out of 171 participants, 127 participants’ 10-m gait data was used to train the classification model (Tables 3, 1a). In this sample of participants, 25 (19.7%) individuals experienced at least two falls in a year. The trained models were then blind tested on 44 participants who were followed-up for 6-months for falls (Table 1b, c). The test set (44 participants) was isolated during the whole process of training to serve the purpose of blind testing. In this sample of participants, 9 (20.4%) individuals experience their falls during this period. Participants who fell at 6-month follow-up had less confidence in flexibility and had significantly less intake of vitamin D than their non-falling counterparts at baseline (Table 1c). Additionally, the results indicated 26.6% of falls occurred in the afternoon and 33.3% of falls in the evening, whereas about 13.3% in late night and early morning. About 38.4% of indoor falls occurred in the bedroom and about 15.3% of falls occurred on the stairs while 42% of outdoor falls occurred on sidewalks and about 14% occurred in the yard area.

**Table 1:** (a) Anthropometric characteristics of 127 older adults for training the random forest model, (b) fall status of 171 participants into categories of fallers/ non-fallers and (c) anthropometric characteristics and confidence score (i.e., activity, balance, and confidence score) at baseline of 44 older participants with 6-months follow-up fall frequency data for testing random forest model.

**(a)**			
	Fallers (Falls > 2)		
	Fallers (N = 25)	Non-Faller (N = 102)	
Age [years] (mean ± SD)	75.44 ± 8.70	75.77 ± 7.63	
Height [cm] (mean ± SD)	163.8 ± 22.17	149.6 ± 35.32	
Weight [lbs] (mean ± SD)	169.9 ± 40.47	165.4 ± 43.63	

(b)			
		Fallers (Falls > 2)	
	Total participants	Fallers	Non-Fallers
Training data set	127	25	102
Testing data set (6 months follow-up)	44	9	35

**(c)**			
	Fallers (Falls > 2)		
	Non-Faller (N = 35)	Faller (N = 9)	*p*-value
Age [years] (mean ± SD)	73.0 ± 8.1	75.8 ± 9.4	0.14
Height [cm] (mean ± SD)	163.7 ± 14.0	157.6 ± 17.1	0.26
Weight [lbs] (mean ± SD)	165.2 ± 45.02	158.38 ± 60.6	0.33
**Activity Balance and Confidence score (Max 100%) (mean ± SD)**			
Walking outside house	87.0 ± 18.8	86.6 ± 19.3	0.55
Walking on stairs	77.0 ± 26.5	63.3 ± 35.0	0.17
For bending	78.8 ± 29.6	74.4 ± 25.5	0.25
Reaching object	94.7 ± 11.0	81.1 ± 22.0	< 0.01*
Standing on tiptoes	74.4 ± 31.3	76.6 ± 25.0	0.54
Standing on chair	53.5 ± 37.7	50.0 ± 44.1	0.97
In sweeping	86.8 ± 30.1	74.4 ± 36.4	0.11
Walk to car	86.4 ± 17.7	83.3 ± 16.5	0.17
Drive car	90.5 ± 14.7	86.6 ± 23.9	0.10
Walking in parking lot	81.7 ± 27.6	85.5 ± 22.9	0.93
On ramp	84.2 ± 24.1	75.5 ± 29.2	0.15
Walking in crowded places	81.4 ± 22.2	74.4 ± 26.0	0.25
Bumped	72.0 ± 26.4	58.8 ± 32.1	0.09
Using escalator with rail	70.6 ± 32.8	56.2 ± 32.4	0.15
Using escalators without rail	43.3 ± 39.6	28.7 ± 32.7	0.24
Vitamin D intake (%)	61.7%	33.3%	< 0.01*
Calcium intake (%)	58%	77%	0.35

ABC score - as such it is bolded.

Linear and non-linear gait and postural stability features of the participants from the training-data-set are already published^27,41^. In these studies, statistically significant increase in postural sway and gait instability were observed for fallers as compared to their non-falling counterparts. Participants were classified as a “faller” if they experienced two or more falls in the past one year. Training data set consisted of 127 participants (Table 1a) and testing data set consisted of 44 participants (Table 1b, c).

### Performance measures

Three machine-learning experiments were conducted with a different strategy (feature engineering (i.e., principal component (PC) analysis) versus no feature engineering) and different data inputs (linear or nonlinear features versus a combination of linear and nonlinear features). Six metrics were used to evaluate the performance: accuracy, specificity, sensitivity, F1 score (harmonic mean of precision and sensitivity), Matthew’s correlation coefficient (MCC)^56,57^, and AUC. AUC, derived from ROC (Receiver Operating Characteristics) curve, has been used to evaluate the predictive ability of learning algorithms. AUC has a higher degree of consistency and discrepancy comparing to accuracy. Huang et.al, demonstrated empirical evaluations and a formal proof to establish that AUC is indeed statistically consistent and more discriminating than accuracy^58^. MCC has been used as a measure of the quality of binary (two-class) classifications in healthcare applications. MCC is a metric to represent a correlation coefficient between the observed and predicted binary classifications.

## Experiment I: Random forest base model development, validation and blind testing

In experiment I, 58 gait parameters (either linear or nonlinear gait variables—please see the Methods section for description of these variables) were used as the input variables of RF classifier. Three hundred sixty-five trees and one feature at each split was used to build the random forest model using the data from 127 participants. The model was then blind tested on the 44 participants. The performance was 71.8 ± 7.0% of accuracy, 53.3 ± 11.5% of sensitivity, and 76.6 ± 11.6% of specificity when using only the linear gait variables. On the other hand, using nonlinear variables, we found an accuracy of 61.4 ± 3.2%, the sensitivity of 86.7 ± 4.7%, and specificity of 54.9 ± 4.8%. While the base model with input as linear variables had good accuracy, low sensitivity limits the clinical utility of this model.

## Experiment II: Random forest model with feature engineering

In the second experiment, we applied the feature engineering process. As the first step, an unsupervised feature selection was applied to remove the potential risk of source discrepancy between training and test dataset. Among 58 variables, four variables were removed from this step. As a result, 32 linear and 22 nonlinear variables were used in the PC analysis. Using 99% variability covering as the guideline, 16 PCs from linear variables and 18 PCs from nonlinear variables were derived, respectively. The RF model on the 16 linear PCs achieved an overall 61.4 ± 3.2% accuracy, 86.7 ± 4.7% sensitivity, 54.9 ± 4.8% specificity. The RF model on the 18 nonlinear PCs achieved an accuracy of 74.8 ± 5.5%, sensitivity of 80.8 ± 11.5% and specificity of 73.4 ± 9.5%. The comparisons of Experiment I and Experiment II on linear gait features and nonlinear gait features are summarized in Table 2 to show the advantages from the feature engineering process.

**Table 2:** (a) Experiment I, II comparison on linear gait variables and (b) Experiment I, II comparison on nonlinear gait variables.

	Accuracy	Sensitivity	Specificity	F-1 score	MCC	AUC
**(a)**						
Experiment I (Base RF Model) Mean ± SE	0.7182 ± 0.0704	0.5333 ± 0.1148	0.7657 ± 0.1157	0.4384 ± 0.0250	0.2765 ± 0.0482	0.6260 ± 0.0143
Experiment II (RF with Feature Engineer) Mean ± SE	0.7159 ± 0.1199	0.6778 ± 0.1610	0.7257 ± 0.1882	0.5044 ± 0.0546	0.3660 ± 0.0696	0.6865 ± 0.0261
**(b)**						
Experiment I (Base RF Model) Mean ± SE	0.6136 ± 0.0321	0.8667 ± 0.0468	0.5486 ± 0.0482	0.4790 ± 0.0177	0.3371 ± 0.0283	0.6435 ± 0.0275
Experiment II (RF with Feature Engineer) Mean ± SE	0.7478 ± 0.0551	0.8000 ± 0.1147	0.7343 ± 0.0953	0.5672 ± 0.0313	0.4541 ± 0.0393	0.7845 ± 0.0113

**Figure 1:**
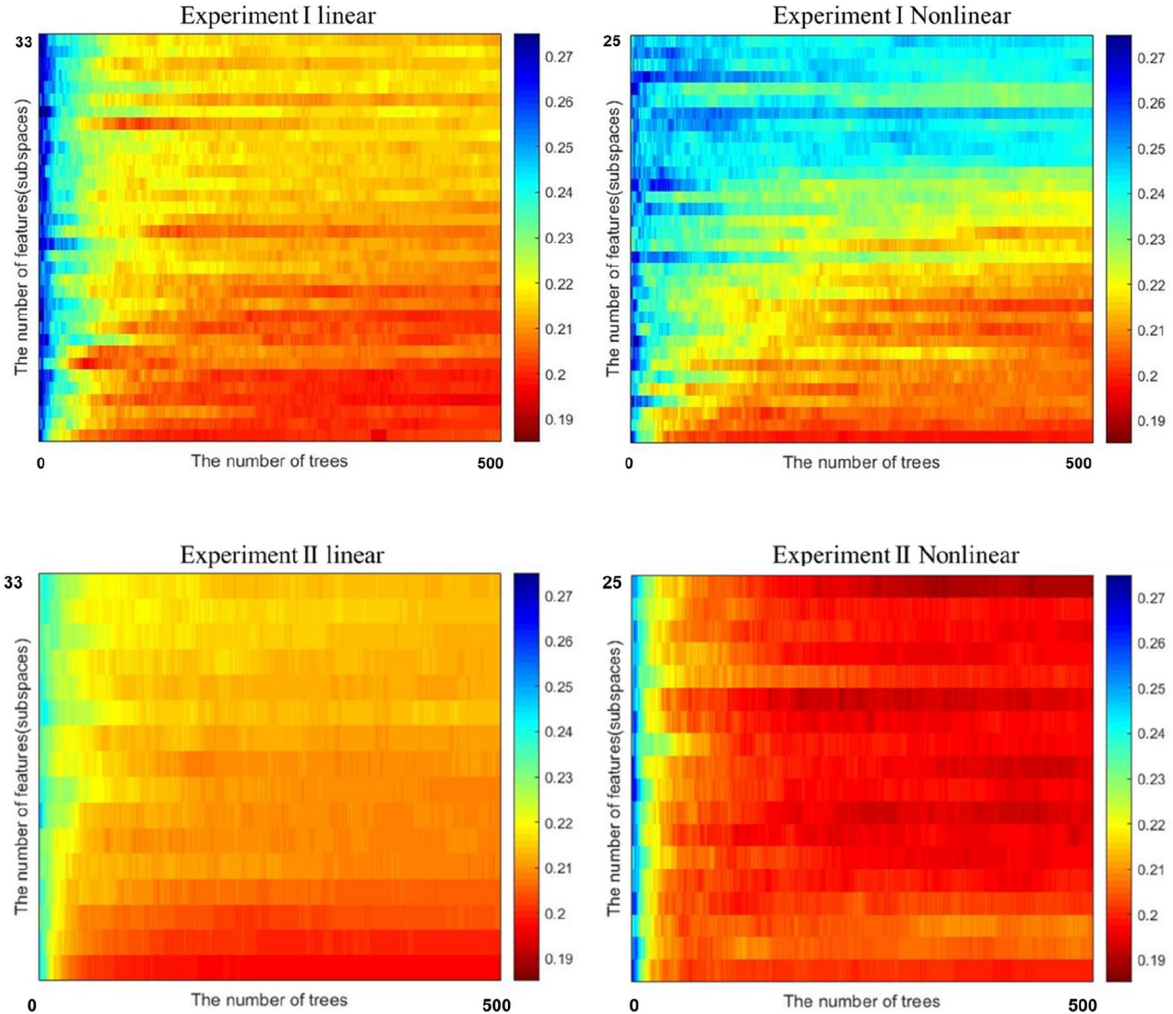
Heatmap of errors from Experiment I (left) and Experiment II (right). The X-axis represents number of tree (0–500) and Y-axis represents number of linear (n = 33) and nonlinear (n = 25) features.

As can be seen in Fig. 1, the heatmap from the second experiment shows a higher concentration of red (low errors) than the first experiment. This demonstrates the value of feature engineering.

## Experiment III: Random forest model with feature engineering and both linear and nonlinear variables

**Figure 2:**
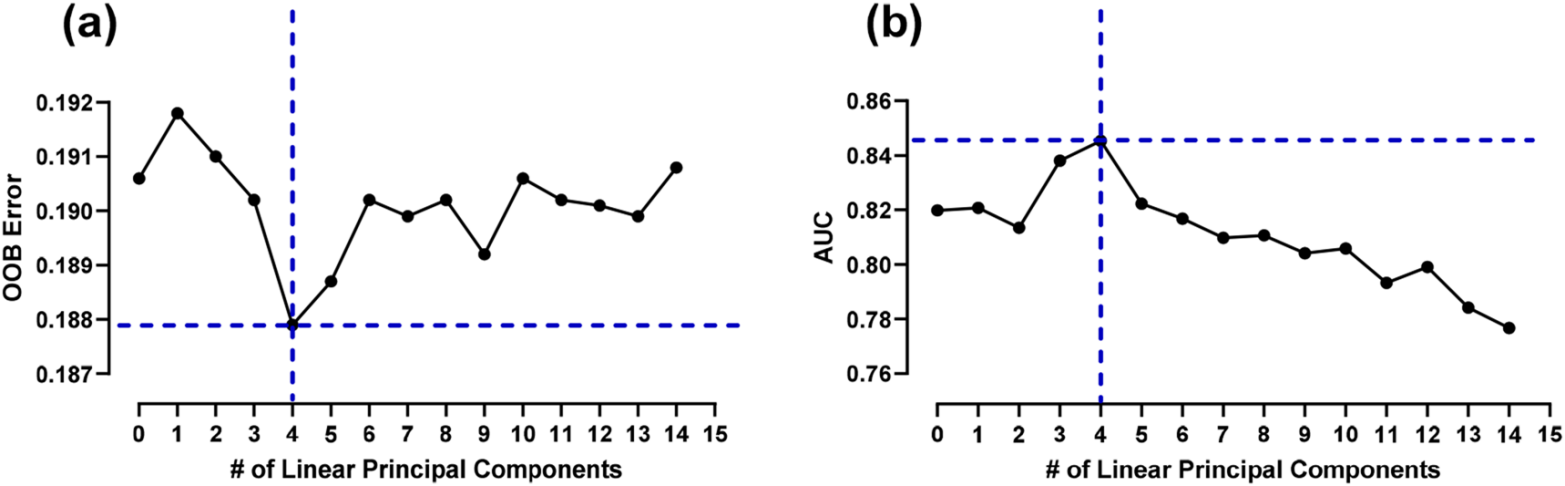
(**a**) OOB error is lowest when 4 linear features are added to model developed using nonlinear gait variables and (**b**) AUC is highest when 4 features are added to the nonlinear variable RF model.

**Figure 3:**
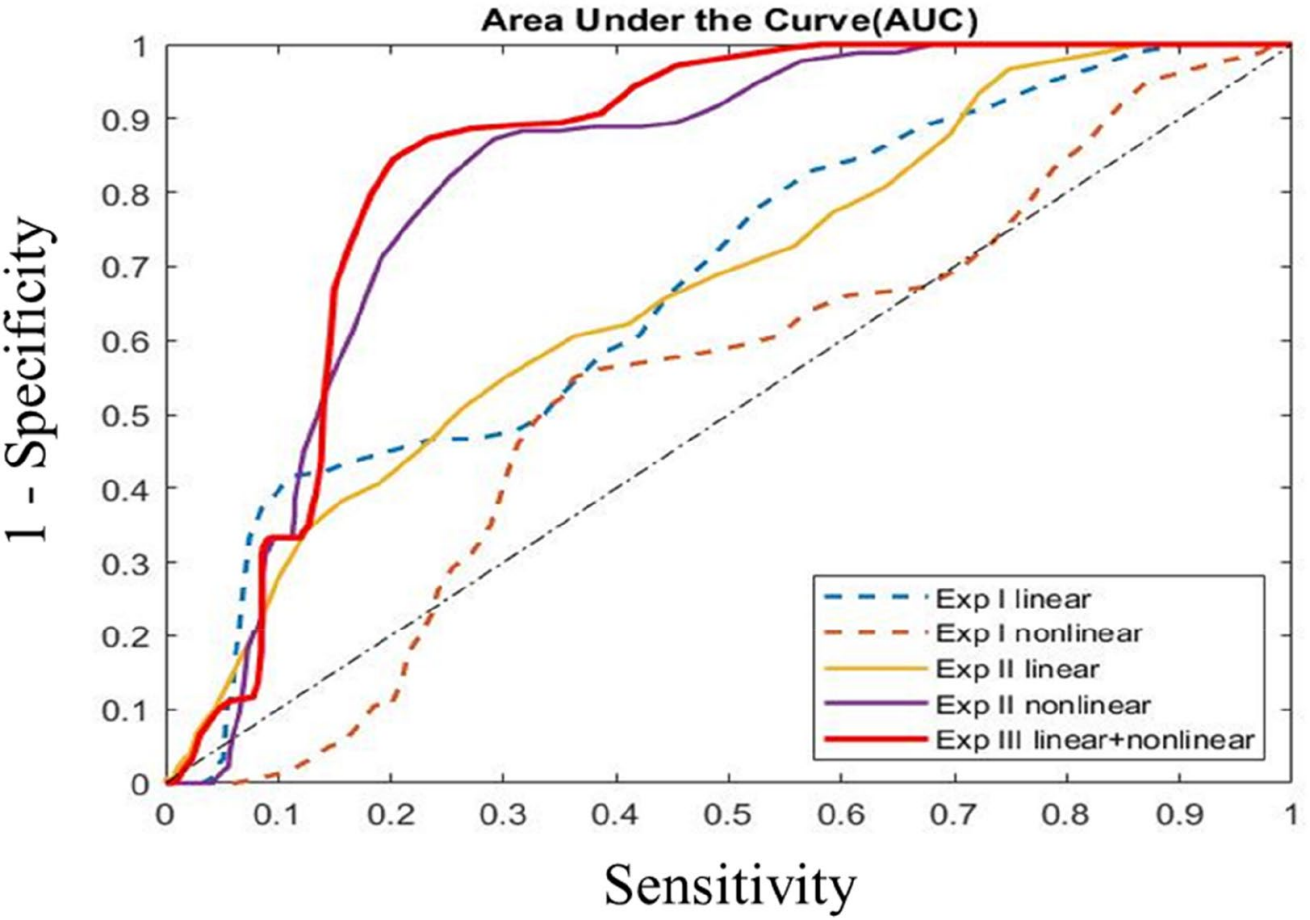
ROC curves depicting AUC from all three experiments I, II, and III. AUC for the three sets of experiments, clearly showing the outperformance from the RF model on linear and nonlinear PCs.

The first two experiments explored the predictive models on linear, and nonlinear variables, independently. We hypothesized the model performance may improve by joining the linear and nonlinear variables. Built upon the RF model on the nonlinear PCs, this experiment was conducted to add linear PCs gradually to assess the performance improvements (see Fig. 2). The elbow point was identified as the number of linear PCs being four from both out-of-bag (OBB) metric (Fig. 2a) and AUC metric (Fig. 2b). The best performing model had an overall 81.6 ± 0.7% accuracy, 86.7 ± 0.5% sensitivity, and 80.3 ± 0.2% specificity (Fig. 3). Ten different random forest runs (using 10 seed values) were used to compute standard error and confidence interval (Appendix Table A5).

### Gait features relevant to fall prediction

**Figure 4:**
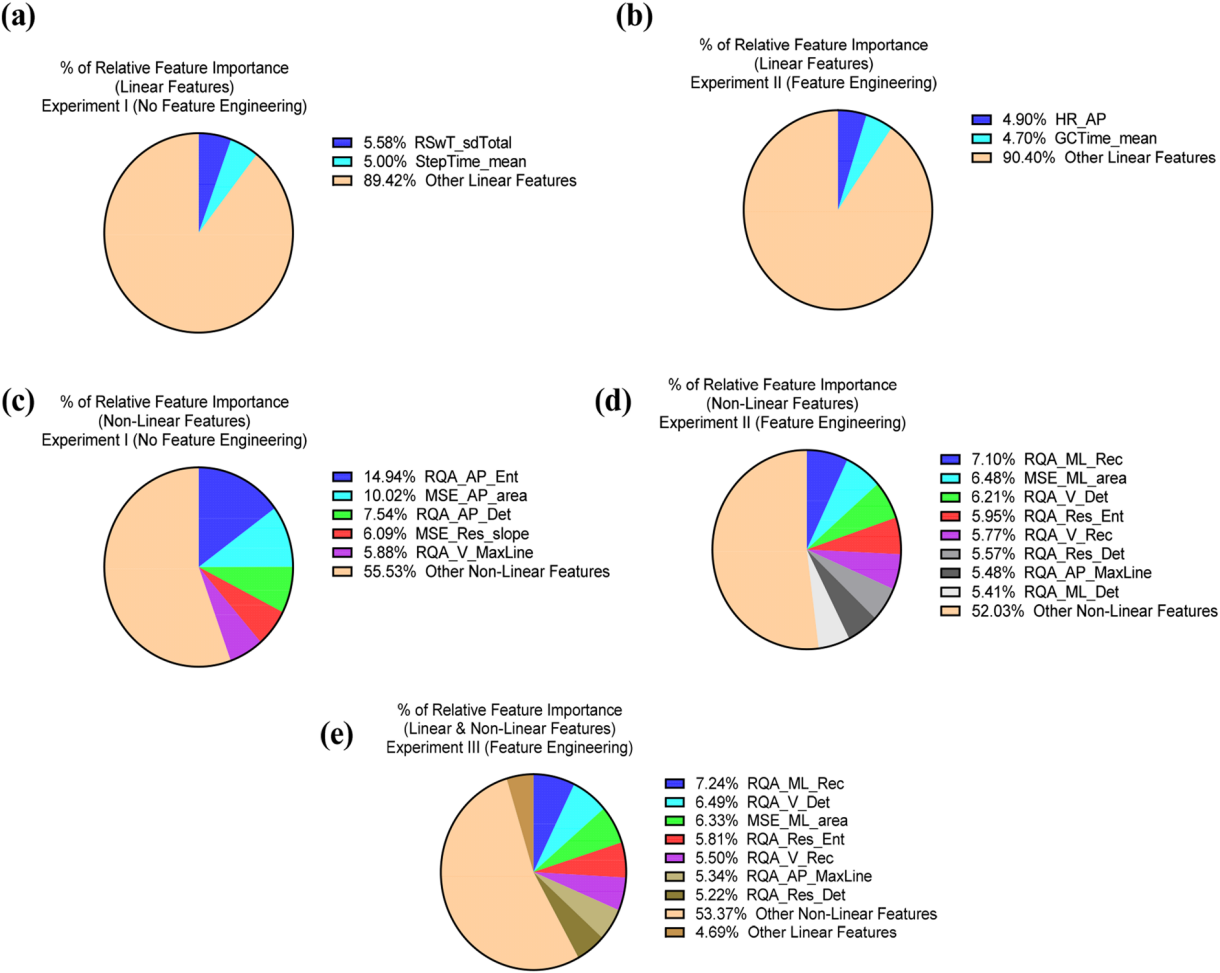
(**a**) linear features with high relative importance in experiment I, (**b**) linear features with high relative feature importance in experiment II with feature engineering, (**c**) Nonlinear features with high relative feature importance in experiment I, (**d**) Nonlinear features with high relative feature importance in experiment II and (**e**) a combination of linear and nonlinear features with high relative feature importance in experiment III.

We implemented nonlinear along with linear features of gait and found that measures such as MSE ^59^ and RQA, which do not require step detection, were significant predictors of fall risk. Using short-distance walking performance test (10-m walking/gait data) afforded by a wearable IMU sensor had discriminative abilities for classification of future fall risk. Additionally, the high predictive performance of random forest classifiers revealed important gait features relevant to fall prediction modeling using both linear and nonlinear gait variables. Indeed, recurrence (RQA_ML_Rec) and complexity (MSE_ML_area), along with determinism (RQA_V_Det) and recurrence (RQA_V_Rec) and, overall walking time series complexity (RQA_Res_Ent) while walking were the strongest predictors for discriminating high versus low fall risk in the older adults. Entropy, a measure of gait complexity, was further identified as a critical predictor for discriminating high versus low fall risk (Fig. 4). Additionally, linear gait characteristics such as step-time and swing-time, as well as smoothness of gait as measured by harmonic ratio had the high predictive performances.

### Out-of-bag estimate of performance

In machine learning, algorithms are tuned to identify the best parameters by using cross-validation data set^60^. RF performs a type of cross-validation in parallel with the training step by using Out of Bag (OOB) samples^61^. OOB is a method of measuring the classification errors of RF. In the process of training, each tree is grown using particular bootstrap samples. Since bootstrapping is sampling with replacement from the training data, some of the participants will be left-out of the sample, while some participant data will be repeated in the sample. The left-out participants, constitute OOB sample. On average, each tree is grown using 2/3 of training participants^62^, leaving 1/3 as OOB. Since OOB participants were not used in the tree construction, they can be used to estimate the ensemble prediction performance. The ensemble classification error can be computed by aggregating only its OOB errors as:

1 $$ER \approx ER^{OOB} = n^{ - 1} \mathop \sum \limits_{i = 1}^{n} I\left( {\hat{Y}^{OOB} \left( {X_{i} } \right) \ne Y_{i} } \right)$$

An out-of-bag estimate of the classification performance helps improve the generalizability of models by evaluating predictions on those observations which were not used in the building of the tree. The OOB errors were computed by an average of 10 runs, since each RF model was built using 10 different random seeds. Table 3 below presents OOB errors for the best performing model from each experiment. The results indicate that combining both the linear as well as nonlinear gait parameters into the model will likely have less OOB errors—thus, a better prediction model.

**Table 3:** OOB errors, number of trees and number of features at each split are tabulated for both experiment I, II, III.

	OOB error	Number of trees	Number of random features at each split
Exp I (Linear)	0.1953	365	1
Exp II (Linear)	0.1969	230	1
Exp I (Non-Linear)	0.1909	311	3
Exp II (Non-Linear)	0.1894	490	18
Exp III (Non-Linear + Linear)	0.1879	490	20

## Discussion

The present study investigated capabilities of using a wearable sensor and, extracted linear and nonlinear gait variables along with a machine learning approach to predict fall risks among community dwelling older adults. The results indicate that the use of both linear and nonlinear gait variables can increase fall risk prediction accuracy, sensitivity, and specificity using a Random Forest Classifier. Fall risk assessment methods estimate the probability of future falls through the identification of predictive fall risk factors^63–65^. This process is a critical first step before employing preventative and amenable intervention strategies. As it stands, traditional fall risk assessments conducted in community-dwelling settings rely on qualitative and subjective assessments that lack the predictive power to assess prospective fall incidence accurately and reliably. Spurred by this gap, gait assessment has become a prevalent fall risk tool, with researchers reporting a spate of gait-specific risk factors^66^.

For older adults, a linear analysis of gait identifies variability as the resulting errors brought upon by internal and external stressors, whereby the amount of variability delineates impaired gait. Studies ascertained that an individual’s inability to walk in a repetitive and stable manner as a possible sign of an evolving gait disorder leading to falls^5^. Furthermore, gait intracycle variability among older adults without any noticeable gait impairment may reveal the gradual deterioration of stability mechanics during gait. As such, nonlinear analysis of gait identifies variability as the global health of the physiological system^67^. Where traditionally the amount of variability reflects less stability, in this context, the underlying structure of variability reflects adaptive capacity in the framework of fall risk^68^. In other words, feedback mechanisms associated with locomotor control system can be characterized by regularity in the time-series data (weak or degraded neuromuscular system being characterized by increased regularity). This analysis provides essential insights into the dynamic stability of walking, a useful tool for evaluating and quantifying gait deficits associated with fall prone individuals. Thus, older adults’ fall risk models utilizing gait variables using an IMU must consider the organization of variability as well as the fluctuations occurring across all time scales utilizing nonlinear dynamics.

In terms of classical concepts of physiological control and homeostasis^67^, healthy systems are self-maintained or regulated to reduce variability and maintain physiologic constancy. However, the output of a wide variety of systems such as gait, fluctuates in a complex manner. Age-related deterioration of sensory and neuromuscular control mechanisms is not adequately identified through linear analysis techniques, as most of these measures rely on accurate step detection—a notoriously difficult event to detect with a single IMU given the reported decrements in older adult gait, i.e. “shuffling” and asymmetrical gait. Nonlinear measures are not bound by algorithmic event detection techniques. They are expressed as time series trajectories that observe the evolution of the locomotor control system, in which a degraded neuromuscular system can be identified by increased entropy in the physiological time series. Understanding this gait complexity via nonlinear scaling techniques may reveal the presence of long-range, power-law correlation that may describe subtle changes in health and may be able to provide cogent measures of physiologic control. As such, we hypothesized that these nonlinear regulatory systems (e.g., gait) are operating far from equilibrium and that maintaining constancy is not the goal of physiologic control. Understanding this relationship may provide new approaches to assessing a variety of health risks and predict adverse health conditions and outcomes.

We found feature engineering increased sensitivity of classification from 53.3 ± 11.5 to 86.7 ± 4.7%, however decreased accuracy from 71.8 ± 7.0 to 61.4 ± 3.2% when using gait derived linear features (Table 4A). But with the addition of non-linear features, feature engineering exceeded in classification performance with an accuracy of 74.8 ± 5.5%, sensitivity of 80.8 ± 11.5% and specificity of 73.4 ± 9.5% (Table 4B). Thus, our results justify the importance of nonlinear variables in addition to linear variables for increasing the prediction ability of the model, thus delivering the most important advances in this research realm. By including both linear and nonlinear feature variables according to their feature importance, the classification model outperformed models created solely using either linear or nonlinear predictor variables. These findings suggest that nonlinear gait measures are sensitive to a subtle change in dynamic walking stability control among community-dwelling older individuals and, is an essential parameter regulation measure required for an accurate fall risk assessment.

**Table 4:** Gait parameters and their definitions.

Gait parameter	Definition
Gait cycle time (s)	Time elapsed between two consecutive heel contacts of the ipsilateral foot
Single support time (s)	Time elapsed from the heel contact to the toe off of a single footfall
Double support time (s)	Time elapsed from the heel contact of one foot to the toe off of the contralateral foot. It is the sum of two periods of double support in the gait cycle
Swing time (s)	Time elapsed between toe off of a gait cycle to the subsequent heel contact of the same foot
Gait speed (cm/s)	Total distance walked divided by duration of walk
Root mean square (RMS *norm*)	Statistical measure of the trunk acceleration magnitude in the AP, ML, or V direction compared to the total trunk acceleration magnitude
Coefficient of variation (CV)	Measure of variability normalized to the mean of a specific gait parameter. CV = (SD/Mean) × 100

Previous studies have utilized random forest for classification of fall risk^63,69,70^, but lacked external validity as they were limited to postural sway^69^, wrist movements^63^ and, study samples of only one gender^70^. Postural sway measures alone were used along with random forest classification to predict fall risk in multiple sclerosis^69^, predicting falls with an accuracy of 71.2%, sensitivity of 71.4% and specificity of 73.5%, albeit it was not a blinded test. The essence of our current study was to provide background knowledge for a new study incorporating both gait and postural features with potential of higher fall prediction accuracy along with high sensitivity and specificity values. Moreover, to the best of our knowledge, most of literature on fall prediction utilizing machine learning techniques reports cross-validated results only and not of blind testing results. We believe that blinded testing performance will help improve the generalizability and robustness of the model in predicting future falls without having patient’s own training data sets in the baseline data collection realm.

A limitation of the present study is that it only included a single sensor (situated at the trunk) and a test (10-m walk test). We have earlier reported that short gait data sets may be insufficient to produce reliable nonlinear measures^71^. Although linear and nonlinear features extracted from short 10-m gait may not show statistical significance but may have carried important weightages in ML models to discriminate fallers and non-fallers. On the other hand, this helps in developing quick feasible fall risk assessment tests that are feasible to carry out with minimal risk in community living environments. From the clinical perspective, the ability to quantify a patient’s functional capacity in an objective way is attractive, as many clinical tests rely on a subjective assessment. Thus, gait-based machine-learning models may help better understand basic motor health behavior processes and could potentially enhance clinical practice. In conclusion, the study confirmed that older adults who have a high risk of falling have gait control deficits and these deficits can be measured by linear and nonlinear variability analysis of walking timeseries. The novel contribution of this investigation is identifying the importance of linear and nonlinear gait variables that are sensitive to gait impairments in older adults as a function of fall risk. Additionally, wearable technology allowed us to gather data where it matters the most to answer fall-related questions, i.e., community-dwelling environments and not in the gait laboratory. This study opens new prospects of clinical testing using gait stability measures with a wearable sensor that may be relevant for assessing fall risks at home and senior living environments.

## Methods

All methods were performed in accordance with the guidelines of the Declaration of Helsinki, and approved by the Institutional Review Board (or Ethics Committee) of Virginia Tech Institutional review Board (protocol code 11-1088 and 10-04-2013 as the date of approval). The study was conducted in four different community centers in Northern Virginia (Dale City, Woodbridge, Leesburg, and Manassas), using the same set of instruments i.e., Inertial Measurement Unit (IMU) on different days. All participants provided written consent before beginning the study. Participants wore comfortable attire and had to perform a 10 m walk. Ten-meter walk was chosen based on the assumption that at least 10 s of continuous walking activity can be detected during the activity of daily living. The participants were instructed to walk at their normal speed. All participants stood behind the start line quietly for 5 s, the experimenter started data collection and gave an auditory signal “GO” to the participant to start walking at their own normal walking speed. When the participant crossed the 10 m finish line, the participant stood quietly for 5 s until they were asked to come back. The walking trial was repeated twice for all participants. The sampling rate was 100 Hz. Rest of 3 min was provided between each measurement.

### Participants

**Figure 5:**
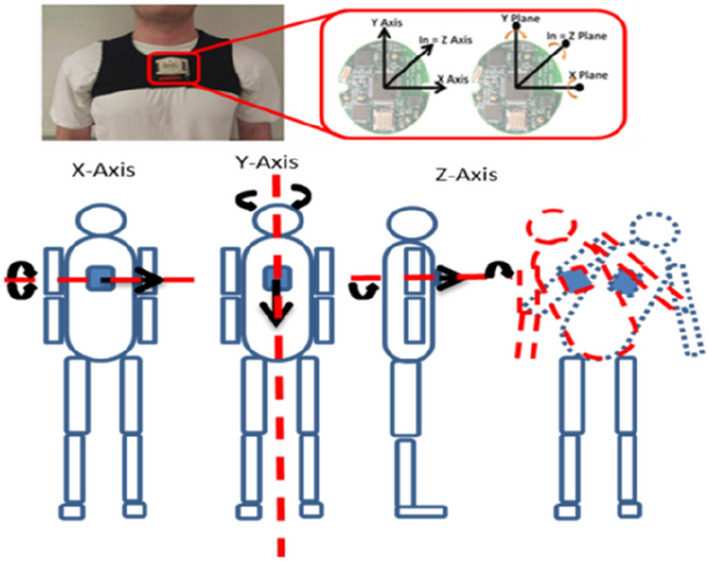
Placement of a wearable IMU system.

A total of 171 older community-dwelling volunteers (age 56–90 years; mean age 74.3 ± 7.6 years) participated in this study. All participants were asked to walk 10 m with one wireless inertial sensor affixed at sternum level (Fig. 5). For the model development, we evaluated 127 community-dwelling older adults’ trunk kinematics using a wearable sensor during walking to unobtrusively assess fall risks that may be amenable to predicting fallers and non-fallers using linear and nonlinear measures. Among the 127 participants, there were 101 non-fallers and 26 fallers. The predictive model accuracy was tested on 44 community-dwelling individuals with six months follow up of their fall history (35 non-fallers vs. 9 fallers). Participants’ history of falls had been recorded for the last 2 years, with emphasis on the frequency and characteristics of falls. Fall history was obtained by self-report, and any subject with at least two falls in the prior year was classified as a faller and the others as non-faller.

### Gait feature extractions

**Figure 6:**
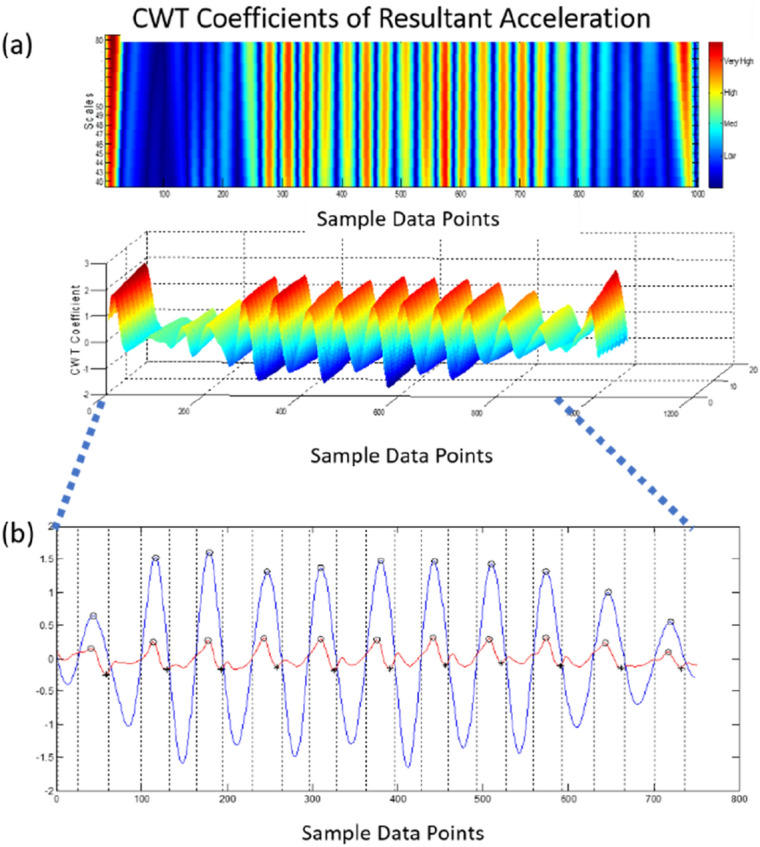
Detection of HC events using the CWT differentiation method. Peaks (blue) equate to HC events; the local minima in the AP acceleration (red) equate to TO events.

Trunk accelerations in the anterior–posterior (AP), medio-lateral (ML), and vertical (V) directions were analyzed. Gait event times were identified using an inertial measurement unit (IMU) positioned over the sternum^37^. A modified continuous wavelet transform (CWT) method was utilized as a gait detection algorithm^38^. The wavelet transform supports time–frequency decomposition of non-stationary signals and does not require preprocessing of the signal, making it ideally suited for a peak detection algorithm^38–40^. The resultant acceleration, a signal invariant to axis alignment, was analyzed to mitigate any alignment errors reliant on IMU placement. Furthermore, due to the placement of the inertial sensor, the gaussian (gaus1) mother wavelet was deemed inappropriate for the inertial data^38^. Instead, a symlet (sym4) mother wavelet with an order of 4 and a scale between 35 and 70, was employed over the resultant acceleration signal to detect the instant events^15^. Heel contacts (HC) were identified as the maxima of the CWT differentiated signal (Fig. 6). The toe-off (TO) events were processed by a windowing technique in which the HC points and the subsequent zero crossings of the CWT differentiated signal determined an appropriate window size where the instant of the first minima in the AP acceleration signal was considered as a TO event (Fig. 6)^41,42^. As per the placement of the inertial sensor and the extracted resultant acceleration, the CWT method previously employed, in which the maxima of a further CWT differentiated signal was considered the final contact event, could not be relied upon to determine the TO time.

Moreover, because of the inherent gait deficiencies associated with the community-dwelling older adults and the intermittent “shuffling of gait,” a window detection method was better suited for the extracted signal^41^. Finally, the right and left HC events were designated by the sign of the vertical angular velocity at the instant of the first HC in which every other HC equated to a stride^38^. The signal was preprocessed with a 4th order low pass Butterworth filter and a cutoff frequency of 2 Hz^42,43^.

Trunk acceleration-based measures of gait spatiotemporal and variability parameters have been used extensively to identify gait characteristics in both healthy and pathologic populations and are often used to quantify fall risk^38,41,43^. Gait variability was assessed by the RMS of trunk acceleration components—the anteroposterior (AP), mediolateral (ML), and vertical (V) directions—and by statistical measures of variability from temporal gait parameters: Standard deviation (SD) and coefficient of variation (CV). CV denotes the variability of a specific gait parameter normalized to its mean value; it is represented as a percentage (CV = SD/mean × 100). The first and last stride during the initiation and termination of gait were excluded from analysis; the local average and the local SD of each time series was computed for each spatiotemporal parameter, as well. Table 4 provides further operational definitions for each parameter.

The normalized RMS of trunk acceleration was implemented to distinguish the proportion of trunk acceleration variability in a particular direction compared to the total acceleration variability. The RMS norm is a normalization method to mitigate the correlation with walking speed^44^. To compute the RMS norm of the trunk acceleration, the RMS of each acceleration component is divided by the vector norm of all the components (AP, ML, V). Furthermore, scaling behavior of walking patterns were assessed.

Harmonic ratio (HR) was computed by decomposing the AP, ML and V acceleration signals into harmonics using discrete Fourier transformation^45^. For HR, the summed amplitudes of the first 10 even harmonics were divided by the sum of amplitudes of the first 10 odd harmonics for the AP and V directions and vice versa for ML acceleration. Since AP and V have two periods every stride, showing dominance of second harmonics and subsequent even harmonics, whereas, ML accelerations have only one period per stride, reflecting a dominance of the first and subsequent odd harmonics^45^. Higher HR is an indication of increased smoothness of gait.

Approximate entropy (ApEn) quantifies the ensemble amount of randomness, or irregularity^46^, contained in a physiological time series. It uses a moving window procedure to determine the probability that short sequences of data points are repeated (within a defined tolerance) throughout the time-series. Here, we use ApEn to quantify the regularity of 3-D trunk accelerations during walking in community-dwelling older adults. Previous research reports that ApEn can be used to detect subtle changes in signal variability that are averaged out in traditional statistical measures of gait stability^33,47^. The algorithm for estimating ApEn was first reported by Pincus^48^.

Sample entropy (SaEn) indexes the regularity of a time series by calculating the probability that having repeated itself for a window length *m*, it will remain similar for *m* + 1 data points, excluding any self-matches and within a matching tolerance *r*. Greater SaEn values delineate irregularity, in which a set of similar points are considered unique as they will likely not be followed by a similar set of matching points within a specified tolerance. Higher values are considered part of a healthy, robust system able to adapt to challenges and unexpected perturbations. Lower values of SaEn are associated with higher regularity of the time series, in which there is a greater likelihood that datasets of matching epochs in a time series will be followed by another match within a specified tolerance. Lower values denote a possible rigid, disease state—unable to adapt to challenges or walking perturbations. SaEn was computed with the resultant acceleration time series. Parameters *m* and *r* were chosen accordingly obtaining *m* = 2 and *r* = 0.25 for both directions.

Multiscale entropy (MSE) is a regularity measure that quantifies the information content of postural/gait fluctuations over a range of physiologically relevant time scales while sample entropy is computed for every consecutive coarse-grained time series. The entropy values are then plotted as a function of the time scales in which the area under the curve reveals the signal’s complexity index. A complex signal is associated with a time evolution with a rich structure on multiple scales. For white noise, which is irregular on small time scales but not structurally complex, the entropy decreases for larger time scales. For a complex signal, such as pink 1/f noise, the entropy remains high on different scales. For the computation of MSE the input parameters m = 2 and r = 0.25 were chosen similar to the SaEn algorithm.

Recurrence quantitative analysis (RQA): recurrence quantitative analysis is a nonlinear analysis technique^49,50^ recently used in gait signal analysis^50^. The local recurrence of data points during gait in the reconstructed state space allows RQA to quantify deterministic structures and associated non stationarities^51^. In this study, an embedding dimension of 5 and a delay of 10 was chosen^50^. The recurrence plot was made with radius of 40% of the maximum distance and cells below this threshold were identified as recurrent points. RQA measures such as entropy, recurrence, determinism, and MaxLine were computed for this study. All gait descriptors were calculated using custom MATLAB scripts. A list of both linear and nonlinear gait variability descriptors used in this study is provided below (Table 5).

**Table 5:** List of 58 linear and nonlinear gait variability descriptors used in fall classification.

Gait descriptors	Description	Linear (L) nonlinear (NL)
RQA_AP_Ent	Anterior Posterior signal Entropy from Recurrence Quantification Analysis	NL
MSE_AP_area	Anterior Posterior signal Multiscale Entropy using Area algorithm	NL
RSwT_sdTotal	Standard deviation of swing time (Right Foot)	L
StepTime_mean	Average Step Time	L
RSwt_cv	Coefficient of variation of swing time (right foot)	L
Velocity	Walking velocity	L
LSwT_cv	Coefficient of variation of swing time (left foot)	L
DST_cv	Coefficient of variation of double support time	L
LSST_mean	Mean single stance duration (left foot)	L
Time2FirstQuartile_Velocity	Time taken to reach first quartile of walking velocity	L
Time2Median_Velocity	Time taken to reach median of walking velocity	L
RMS_AP	Anterior posterior signal root mean square	NL
DST_sdTotal	Standard deviation of double support time	L
HR_ML	Harmonic ration in medial–lateral direction	L
RMS_ML	Medial lateral signal root mean square	L
Time2ThirdQuartile_Velocity	Time taken to reach third quartile of walking velocity	L
RQA_ML_MaxLine	Medial lateral signal MaxLine from recurrence quantification analysis	NL
GCTime_cv	Coefficient of variation of gait cycle time	L
RQA_Res_MaxLine	Resultant signal MaxLine from recurrence quantification analysis	NL
RSST_mean	Mean single stance duration (right foot)	L
RQA_V_MaxLine	Vertical signal MaxLine from recurrence quantification analysis	NL
MSE_AP_slope	Anterior posterior signal multiscale entropy using slope algorithm	NL
LSwT_mean	Mean of swing time (left foot)	L
RQA_AP_Det	Anterior posterior signal determinism from recurrence quantification analysis	NL
MSE_ML_area	Medial lateral signal multiscale entropy using area algorithm	NL
StepTime_sdTotal	Standard deviation of step time	L
RQA_V_Rec	Vertical signal recurrence from recurrence quantification analysis	NL
RSST_sdTotal	Standard deviation of single stance duration (right foot)	L
MSE_Res_slope	Resultant signal multiscale entropy using slope algorithm	NL
RQA_AP_Rec	Anterior posterior signal recurrence from recurrence quantification analysis	NL
RMSR_V	Vertical signal normalized root mean square	L
DST_mean	Average double support time	L
GCTime_sdTotal	standard deviation of gait cycle time	L
LSwT_sdTotal	Standard deviation of swing time (left foot)	L
RMSR_AP	Anterior posterior signal normalized root mean square	L
RQA_V_Ent	Vertical signal entropy from recurrence quantification analysis	NL
MSE_Res_area	Resultant signal multiscale entropy using Area algorithm	NL
RQA_ML_Rec	Medial lateral signal recurrence from recurrence quantification analysis	NL
LSST_sdTotal	Standard deviation of single stance duration (left foot)	L
StepTime_cv	Coefficient of variation of step time	L
HR_V	Harmonic ration in vertical direction	L
RSwT_mean	Mean of swing time (right foot)	L
RMSR_ML	Medial lateral signal normalized root mean square	L
MSE_ML_slope	Medial lateral signal Multiscale entropy using slope algorithm	NL
MSE_V_slope	Vertical signal multiscale entropy using slope algorithm	NL
GCTime_mean	Mean of gait cycle time	L
RMS_V	Vertical signal root mean square	L
MSE_V_area	Vertical signal multiscale entropy using area algorithm	NL
RQA_Res_Ent	Resultant signal entropy from recurrence quantification analysis	NL
RQA_Res_Det	Resultant signal determinism from recurrence quantification analysis	NL
RQA_AP_MaxLine	Anterior posterior signal MaxLine from recurrence quantification analysis	NL
RQA_V_Det	Vertical signal determinism from recurrence quantification analysis	NL
RSST_cv	Coefficient of variation of single stance duration (right foot)	L
RQA_ML_Det	Anterior posterior signal determinism from recurrence quantification analysis	NL
RQA_ML_Ent	Medial lateral signal entropy from recurrence quantification analysis	NL
LSST_cv	Coefficient of variation of single stance duration (left foot)	L
RQA_Res_Rec	Resultant signal recurrence from recurrence quantification analysis	NL
HR_AP	Harmonic ration in anterior posterior direction	L

### Random forest predictive model

**Figure 7:**
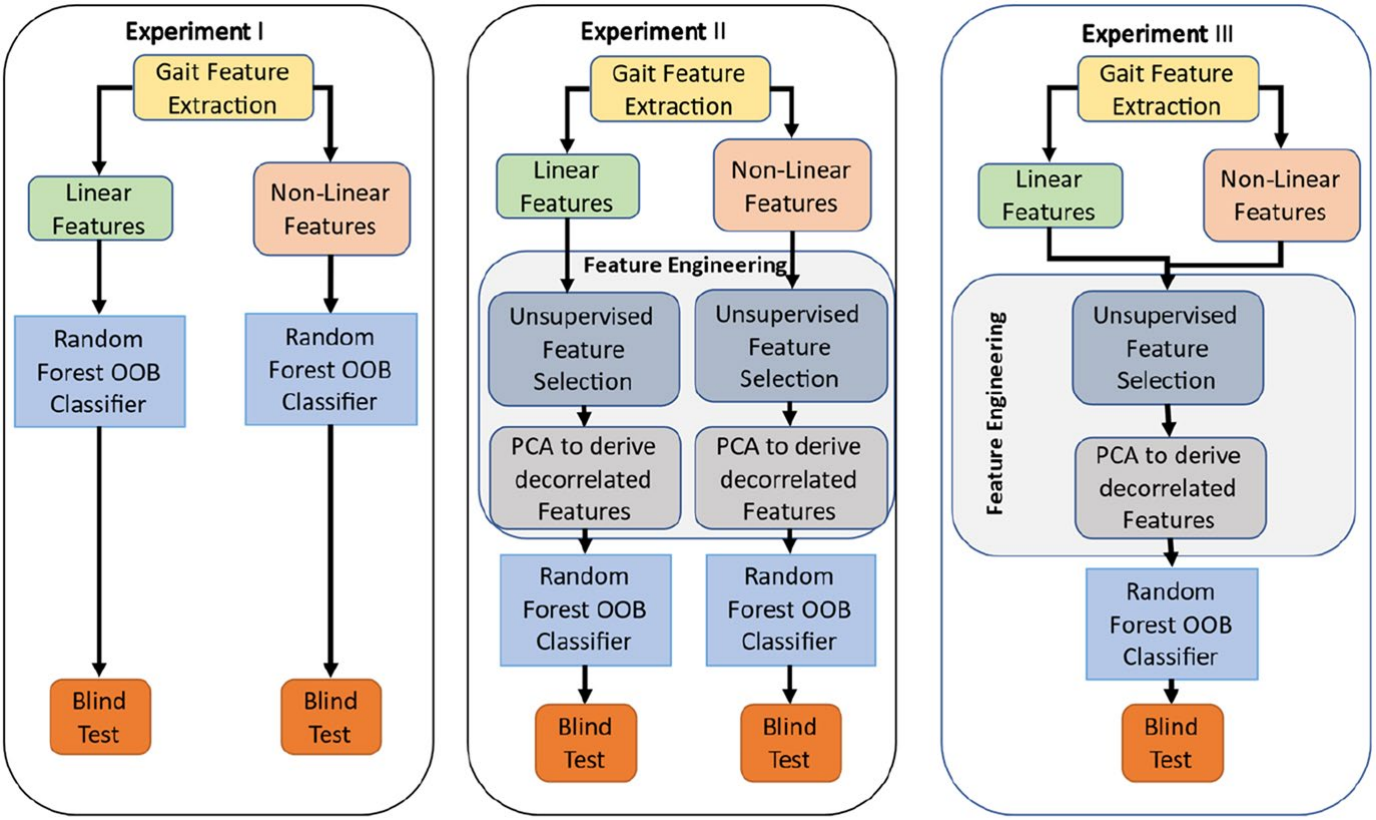
Workflow of three designed experiments (OOB: Out of Bag RF strategy).

In this study, we conducted three experiments for predictive model development and validation using random forest (RF)^53^, a well-studied supervised machine learning algorithm as the classifier. RF creates the forest with a number of trees. With more trees in the forest, it is more likely to provide robust predictions with high accuracy^54^. Each decision tree is created from randomly chosen features and test-data participants and utilizing a set of rules to predict fall risk. Finally, votes are calculated for each predicted output from decision trees, and majority voting is considered a final prediction. Some advantages of RF are that it can handle missing values^55^, and it provides robust prediction without overfitting^54^. As seen in Fig. 7, Experiment I explores the applicability of RF on all 58 gait parameters (both linear and nonlinear—Table 5) trained on 127 participants and blind tested on the 44 subjects. In Experiment II, our focus was to employ two feature engineering steps to improve the RF classifier. The first step was unsupervised feature selection. Two sample *t* test was applied to evaluate the potential risk of source discrepancy in gait parameters using a training dataset. Specifically, the trained data was randomly split into two groups, and the *p *value for each variable was used to evaluate the risk factor of each gait parameters. This procedure was repeated *n* (= 1000) times. The averaged *p *values represented the ranked potential risk of source discrepancy for each predictor. The second step applied principal component analysis (PCA) to orthogonalize the original features into less correlated principal components (PCs). Because the gait features are derived from 10 m walk with dynamic motion, inherently, the features may have similar characteristics. One of the limitations of having highly correlated features is the trained RF may be destabilized which will weaken its clinical value. We hypothesized PCA approach may address this issue. Usually, a few PCs may sufficiently account for most of variability in the original feature space. In Experiment II, PCs capturing 99% of the variability in the original dataset were derived for the RF classifier. Experiment I and II used linear and nonlinear features independently to assess the contributions from feature engineering. Experiment III was then conducted on RF model in conjunction with feature engineering using combined linear and nonlinear features.

## Supplementary Information

Supplementary Information.
