# Supplementary material for: Prediction of fall risk among community-dwelling older adults using a wearable system
Source: Sci Rep. 2021 Oct 25;11:20976. doi: 10.1038/s41598-021-00458-5 (PMC8545936; doi:10.1038/s41598-021-00458-5)
Supplement: Supplementary file 1 — Supplementary Information. [file 41598_2021_458_MOESM1_ESM.docx]

**Appendix**

Table A1: Information about participant’s fall status and its change in follow-up examination after six months

| **Subject #** | **Initial Status** | **# Falls** | **Follow Up Status**  **(After 6 months)** | **# Falls** | **Fall Status Change** |
| --- | --- | --- | --- | --- | --- |
| SC17 | 0 | 0 | 1 | 5 | 1 |
| SC21 | 0 | 0 | 0 | 0 | 0 |
| SC05 | 1 | 50 | 1 | 8 | 0 |
| SC20 | 1 | 10 | 1 | 6 | 0 |
| MC31 | 0 | 0 | 0 | 0 | 0 |
| MC01 | -- | -- | 0 | 0 | -- |
| MC03 | -- | -- | 0 | 0 | -- |
| MC04 | -- | -- | 0 | 0 | -- |
| MC07 | -- | -- | 0 | 1 | -- |
| MC08 | -- | -- | 0 | 1 | -- |
| MC10 | -- | -- | 0 | 0 | -- |
| MC11 | -- | -- | 0 | 1 | -- |
| MC12 | -- | -- | 0 | 1 | -- |
| MC13 | -- | -- | 0 | 0 | -- |
| MC16 | -- | -- | 0 | 0 | -- |
| MC17 | -- | -- | 0 | 1 | -- |
| MC20 | -- | -- | 0 | 1 | -- |
| MC21 | -- | -- | 0 | 0 | -- |
| MC22 | -- | -- | 0 | 0 | -- |
| RC01 | 0 | 0 | 0 | 0 | 0 |
| RC04 | 1 | 3 | 0 | 1 | 2 |
| RC06 | 0 | 0 | 0 | 0 | 0 |
| RC07 | 0 | 0 | 0 | 0 | 0 |
| RC08 | 0 | 0 | 0 | 0 | 0 |
| RC10 | 0 | 1 | 0 | 0 | 0 |
| RC11 | 0 | 0 | 0 | 0 | 0 |
| RC13 | 0 | 0 | 0 | 1 | 0 |
| RC14 | 0 | 0 | 0 | 0 | 0 |
| RC16 | 0 | 0 | 0 | 0 | 0 |
| RC17 | 0 | 0 | 0 | 0 | 0 |
| RC22 | 0 | 1 | 0 | 1 | 0 |
| RC23 | 0 | 1 | 0 | 0 | 0 |
| RC25 | 0 | 0 | 0 | 0 | 0 |
| RC26 | 1 | 3 | 1 | 2 | 0 |
| RT01 | 0 | 0 | 0 | 1 | 0 |
| RT03 | 1 | 11 | 1 | 3 | 0 |
| RT18 | 1 | 2 | 0 | 1 | 2 |
| RT22 | 0 | 0 | 0 | 0 | 0 |
| LS01 | 0 | 0 | 0 | 0 | 0 |
| LS02 | 0 | 0 | 0 | 0 | 0 |
| LS05 | 1 | 2 | 0 | 0 | 2 |
| LS06 | 0 | 0 | 0 | 0 | 0 |
| LS08 | 0 | 0 | 0 | 0 | 0 |
| LS10 | 0 | 0 | 0 | 0 | 0 |

Table A2: Information about time of day when the fall occurred in participants

| Early Morning | Mid-Morning | Around Noon | After Noon | Evening | Night |
| --- | --- | --- | --- | --- | --- |
| 13.3% | 6.6% | 6.6% | 26.6% | 33.3% | 13.3% |
|  |  |  |  |  |  |

Table A3: Indoor falls in participants

| Indoor falls | | | | | |
| --- | --- | --- | --- | --- | --- |
| **Bedroom** | **Bathroom** | **Kitchen** | **Living Area** | **Stairs** | **Other Places** |
| 38.4% | 7.6% | 7.6% | 7.6% | 15.3% | 23% |

Table A4: Outdoor falls in Participants

|  |  | Outdoor Falls |  |  |  |
| --- | --- | --- | --- | --- | --- |
| **Driveway** | **Parking Lot** | **Sidewalk** | **Yard** | **Stairs** | **Others Places** |
| <1% | <2% | 42% | 14% | <2% | 40% |

**Table A5:** Ten different random forest runs (using 10 seed values) were used to compute standard error and Confidence interval. Mean standard error and confidence intervals are reported below.

|  | **Exp I (Linear)** | |  |  |  |  |
| --- | --- | --- | --- | --- | --- | --- |
| **Runs** | **Accuracy%** | **Sensitivity%** | **Specificity%** | **F-1 Score%** | **MCC%** | **AUC%** |
| 1 | 75.00 | 44.44 | 82.86 | 42.11 | 26.28 | 59.84 |
| 2 | 77.27 | 44.44 | 85.71 | 44.44 | 30.16 | 62.38 |
| 3 | 79.55 | 44.44 | 88.57 | 47.06 | 34.53 | 63.65 |
| 4 | 77.27 | 44.44 | 85.71 | 44.44 | 30.16 | 63.49 |
| 5 | 77.27 | 44.44 | 85.71 | 44.44 | 30.16 | 63.65 |
| 6 | 63.64 | 55.56 | 65.71 | 38.46 | 17.62 | 61.11 |
| 7 | 61.36 | 77.78 | 57.14 | 45.16 | 28.17 | 64.44 |
| 8 | 72.73 | 55.56 | 77.14 | 45.45 | 28.91 | 63.65 |
| 9 | 61.36 | 66.67 | 60.00 | 41.38 | 21.60 | 62.06 |
| 10 | 72.73 | 55.56 | 77.14 | 45.45 | 28.91 | 61.75 |
| Mean | 71.8 | 53.3 | 76.6 | 43.8 | 27.7 | 62.6 |
| SD | 7.0 | 11.5 | 11.6 | 2.5 | 4.8 | 1.4 |
| CI | (67.4-76.1) | (46.2-60.4) | (69.3-83.7) | (42.2-45.3) | (24.6-30.6) | (61.7-63.4) |

|  | **Exp II (Linear)** | |  |  |  |  |
| --- | --- | --- | --- | --- | --- | --- |
| **Runs** | **Accuracy%** | **Sensitivity%** | **Specificity%** | **F-1 Score%** | **MCC%** | **AUC%** |
| 1 | 68.18 | 66.67 | 68.57 | 46.15 | 29.19 | 68.57 |
| 2 | 79.55 | 66.67 | 82.86 | 57.14 | 44.85 | 69.52 |
| 3 | 79.55 | 66.67 | 82.86 | 57.14 | 44.85 | 73.02 |
| 4 | 77.27 | 55.56 | 82.86 | 50.00 | 35.78 | 68.89 |
| 5 | 79.55 | 55.56 | 85.71 | 52.63 | 39.72 | 65.71 |
| 6 | 72.73 | 66.67 | 74.29 | 50.00 | 34.85 | 69.05 |
| 7 | 79.55 | 44.44 | 88.57 | 47.06 | 34.53 | 67.94 |
| 8 | 50.00 | 88.89 | 40.00 | 42.11 | 24.58 | 63.81 |
| 9 | 50.00 | 100.00 | 37.14 | 45.00 | 32.84 | 68.41 |
| 10 | 79.55 | 66.67 | 82.86 | 57.14 | 44.85 | 71.59 |
| Mean | 71.6 | 67.8 | 72.6 | 50.4 | 36.6 | 68.7 |
| SD | 12.0 | 16.1 | 18.8 | 5.5 | 7.0 | 2.6 |
| CI | (64.1-79.0) | (57.8-77.7) | (60.9-84.2) | (47.0-53.8) | (32.2-40.9) | (67.0-70.2) |

|  | **Exp I (Nonlinear)** | |  |  |  |  |
| --- | --- | --- | --- | --- | --- | --- |
| **Runs** | **Accuracy%** | **Sensitivity%** | **Specificity%** | **F-1 Score%** | **MCC%** | **AUC%** |
| 1 | 59.09 | 88.89 | 51.43 | 47.06 | 32.83 | 62.22 |
| 2 | 61.36 | 77.78 | 57.14 | 45.16 | 28.17 | 62.06 |
| 3 | 68.18 | 77.78 | 65.71 | 50.00 | 35.42 | 67.46 |
| 4 | 63.64 | 88.89 | 57.14 | 50.00 | 37.17 | 60.00 |
| 5 | 63.64 | 88.89 | 57.14 | 50.00 | 37.17 | 68.25 |
| 6 | 59.09 | 88.89 | 51.43 | 47.06 | 32.83 | 63.81 |
| 7 | 56.82 | 88.89 | 48.57 | 45.71 | 30.73 | 62.38 |
| 8 | 59.09 | 88.89 | 51.43 | 47.06 | 32.83 | 66.67 |
| 9 | 61.36 | 88.89 | 54.29 | 48.48 | 34.98 | 64.13 |
| 10 | 61.36 | 88.89 | 54.29 | 48.48 | 34.98 | 66.51 |
| Mean | 61.4 | 86.7 | 54.9 | 47.9 | 33.7 | 64.3 |
| SD | 3.2 | 4.7 | 4.8 | 1.8 | 2.8 | 2.7 |
| CI | (59.3-63.3) | (83.7-89.5) | (51.8-57.8) | (46.8-49.0) | (31.9-35.4) | (62.6-66.0) |

|  | **Exp II (Nonlinear)** | |  |  |  |  |
| --- | --- | --- | --- | --- | --- | --- |
| **Runs** | **Accuracy%** | **Sensitivity%** | **Specificity%** | **F-1 Score%** | **MCC%** | **AUC%** |
| 1 | 79.55 | 77.78 | 80.00 | 60.87 | 50.04 | 79.21 |
| 2 | 63.64 | 100.00 | 54.29 | 52.94 | 44.21 | 77.94 |
| 3 | 72.73 | 77.78 | 71.43 | 53.85 | 40.76 | 76.67 |
| 4 | 79.55 | 77.78 | 80.00 | 60.87 | 50.04 | 79.37 |
| 5 | 79.55 | 77.78 | 80.00 | 60.87 | 50.04 | 80.63 |
| 6 | 75.00 | 77.78 | 74.29 | 56.00 | 43.66 | 78.73 |
| 7 | 72.73 | 77.78 | 71.43 | 53.85 | 40.76 | 77.30 |
| 8 | 79.55 | 66.67 | 82.86 | 57.14 | 44.85 | 77.94 |
| 9 | 68.18 | 100.00 | 60.00 | 56.25 | 48.45 | 78.57 |
| 10 | 77.27 | 66.67 | 80.00 | 54.55 | 41.26 | 78.10 |
| Mean | 74.8 | 80.0 | 73.4 | 56.7 | 45.4 | 78.4 |
| SD | 5.5 | 11.5 | 9.5 | 3.1 | 3.9 | 1.1 |
| CI | (71.3-78.1) | (72.8-87.1) | (67.5-79.3) | (54.7-58.6) | (42.9-47.8) | (77.7-79.1) |

|  | **Exp III linear + nonlinear** | |  |  |  |  |
| --- | --- | --- | --- | --- | --- | --- |
| **Runs** | **Accuracy %** | **Sensitivity %** | **Specificity %** | **F-1 Score %** | **MCC %** | **AUC %** |
| 1 | 81.82 | 88.89 | 80.00 | 66.67 | 58.62 | 80.79 |
| 2 | 81.82 | 77.78 | 82.86 | 63.64 | 53.61 | 80.32 |
| 3 | 79.55 | 88.89 | 77.14 | 64.00 | 55.37 | 79.84 |
| 4 | 81.82 | 88.89 | 80.00 | 66.67 | 58.62 | 81.59 |
| 5 | 81.82 | 88.89 | 80.00 | 66.67 | 58.62 | 82.70 |
| 6 | 81.82 | 88.89 | 80.00 | 66.67 | 58.62 | 81.43 |
| 7 | 81.82 | 77.78 | 82.86 | 63.64 | 53.61 | 80.16 |
| 8 | 81.82 | 88.89 | 80.00 | 66.67 | 58.62 | 81.43 |
| 9 | 81.82 | 88.89 | 80.00 | 66.67 | 58.62 | 80.48 |
| 10 | 81.82 | 88.89 | 80.00 | 66.67 | 58.62 | 80.48 |
| Mean | 81.6 | 86.7 | 80.3 | 65.8 | 57.3 | 80.9 |
| SD | 0.7 | 4.7 | 1.6 | 1.4 | 2.2 | 0.9 |
| CI | (81.1-82.0) | (83.7-89.5) | (79.2-81.2) | (64.9-66.6) | (55.9-58.6) | (80.3-81.4) |
